# 2460 Qualitative study of obesity risk perception, knowledge, and behavior among Hispanic taxi drivers in New York

**DOI:** 10.1017/cts.2018.260

**Published:** 2018-11-21

**Authors:** Aijan Ukudeyeva, Leandro R. Ramirez, Angel Rivera-Castro, Mohammed Faiz, Maria Espejo, Balavenkatesh Kanna

## Abstract

OBJECTIVES/SPECIFIC AIMS: To access obesity risk perceptions, knowledge and behaviors of Hispanic taxi cab drivers and develop a better understanding of the factors that influence health outcomes in this population. METHODS/STUDY POPULATION: Focus groups were conducted at NYC H+H/Lincoln, where subjects were screened and recruited from taxi bases with the help of the local Federation of Taxi Drivers. This was done by utilizing flyers, messages through taxi-base radios, and referrals from livery cab drivers. Approval from the local Institutional Review Board was obtained. The research investigators, developed a structured focus group procedural protocol of open-ended interview questions related to cardiovascular disease. Participants for the focus groups were older than 18 years old and working as livery cab drivers in NYC for at least 6 months. Three focus groups were held with informed consent obtained from each participant in their primary language before the start of each session. After completion of the focus group, participants received a gift voucher for attending the approximately 1-hour session. Focus groups were moderated by trained research staff members at Lincoln. Three main categories of questions were organized based on perception, knowledge, and behavior. Participants were questioned on topics about obesity, CVD and diabetes knowledge; knowledge about etiology, risk perception, possible prevention and interventions. Responses were recorded using audiotapes and transcribed verbatim. If participants did not elaborate on the initial question, a probing question was asked to clarify. The transcript was translated from Spanish by trained bilingual staff and analyzed using standard qualitative techniques with open code method. Four research investigators read the transcript separately and formulated concepts, which were then categorized and formulated into dominant themes. These themes were then compared and analyzed with a group consensus to ensure representative data. Once recurring themes emerged and the saturation point was reached, the study concluded, after enrolling 25 participants. The Health Believe Model (HBM) was employed to understand and explain the perceptions and behaviors of taxi drivers. HBM is one of the most widely recognized models and is used to understand, predict and modify health behavior. HBM helps to identify perception of risks of unhealthy behavior, barriers for having healthy behavior, actions taken by patients to stay healthy, self-efficacy and commitment to goals [12]. RESULTS/ANTICIPATED RESULTS: Of the 25 Hispanic livery cab drivers, 92% were male. The majority of taxi drivers that participated in the study were immigrants (96%), with a mean age of 53 years (ranged 21–69), and 92%, were spoke Spanish. In total, 52% participants identified themselves as Hispanic, 20% White, 4% Black, and 20% did not identify their race. Mean body mass index (BMI) was 31 (22.8–38.7) kg/m^2^. In all, 56% were obese and another 40% were overweight. From this sample, 50% had been diagnosed with hypertension and 27% were living with diabetes. In all, 64% had a high school education or higher. Answers provided by the taxi drivers to focus group questions were recorded, reviewed and divided into 8 dominant themes based on concepts that emerged from the focus groups discussions. (a) Focus group study findings: Themes recorded during the focus group discussions, include poor diet, sedentary lifestyle, comorbidities/risk factors, stress, health not being a priority, discipline, education, and intervention. Participants shared their opinions in regards to these themes with minimal differences, making an emphasis on the fact that the nature of their profession was the root cause. Of the themes, the top 3 dominant themes include poor diet, sedentary/lifestyle and comorbidities/risk factors. (1) Diet: The theme “Poor diet” evolved from 151 related concepts that were described by participants. All 25 participants perceived their diet as bad due to eating high-fat meals associated with the cultural food and restaurant chains with lower food prices and ease of car parking. Drivers also reported that they did not have enough time to eat healthy foods based on their long working hours. They say: “comemos muy tarde por que preferimos montar un pasajero” … stating that they preferred to pick up passengers and delay their meals. However, they consider poor diet as the most decisive factor in their increased risk for obesity, diabetes, and hypertension. (2) Life Style: The theme “Sedentary lifestyle” was derived from 147 similar concepts described by participants. They believe that physical inactivity is another leading risk factor for obesity, diabetes, and CVD. The demands of the profession force them to drive more than 10 hours per day. They understand the importance of daily exercise but they admit that at the end of the workday they are too tired to exercise or “stop working” to participate in exercise as this means less money. They also understand that family history of obesity in addition to poor diet increases their risk of obesity, diabetes, and cardiovascular risks. (3) Comorbidity: The theme “Comorbidities” developed from 143 concepts grouped together. Taxi-drivers perceived that obesity complications directly affects many vital organs, such as the kidneys, the heart, and vasculature. Participants perceive obesity as important risk factor for high blood sugar and cholesterol levels. Taxi drivers see an association between their health condition and their work as a taxi driver. However, taxi-drivers reported that they are more concerned about the economic well-being of their families than themselves. Taxi-drivers begin to intervene in their own health only when more serious health conditions related to obesity, diabetes, and hypertension developed. (4) Work Stress: The theme “Stress/other risk factors” was derived from 141 concepts. Taxi-drivers perceive their profession with lack of organization and high-stress levels as one of the leading risk factors contributing to obesity, diabetes, and cardiovascular disease. They also attribute a combination of stressful lifestyle, poor diet, lack of exercise, consumption of alcohol and cigarettes as determining factors in developing negative health outcomes. “One participant says; Tenemos el paquete completo” … we have the entire package. (5) Health as a priority: The theme “Health is not a priority” was derived from 120 concepts based on the cab drivers’ responses. Taxi drivers prioritize their work while their health takes a back seat. They work long shifts as they feel the pressures of financial responsibilities of their family. They admitted lack of intentions to change their behavior and they consider themselves as “hard headed.” Drivers changed their behavior only when serious health conditions develop that require professional medical attention. Taxi drivers explain that the lack of time as being a big factor in pursuing preventative care. (6) Personal Discipline: The theme “Discipline” evolved from 80 concepts derived from the driver’s transcripts. Taxi drivers are aware of their lack of organizational skills in general, especially when it comes to the balance between work and a healthy lifestyle. Taxi drivers recognize that not being disciplined results in the development of their obesity and chronic health conditions. Drivers admit that they do not have a fixed schedule, with no direct supervision, and cannot find the time to go to the doctor or change their behavior. (7) Health Education: The theme “Education” was derived from79 concepts noted from the focus group discussion. Taxi drivers know that their lack of health education is affecting them. With little understanding about the severity of the disease process it is difficult to take proactive measures. They are interested in the development of programs that will educate them about obesity, diabetes and CVD prevention. They want to attend programs that can educate them about prevention of obesity, diabetes, and CVD prevention with strong focus on healthy eating. They understand that this would increase their ability to change their unhealthy behavior. (8) Health interventions: The last major theme “Intervention” was derived out of 71 concepts. When asked about possible interventions that might help them towards healthy behaviors, taxi drivers think that the use of technology as a means of education is very effective. They understand the most direct route to reach them is by cellphone, email, and social media such as Facebook. They also feel that it would be good to use this type of communication to not only to inform them about health issues, but to also educate them directly. (b) Application of Health Behavior Model: We employed the HBM, one of the most utilized and easy to understand health models (18, 20–22) to explain the knowledge, perception, and health behaviors of our study participants. The HBM consist of 6 posits: (1) risk susceptibility, (2) risk severity, (3) benefits of action, and (4) barriers to action, (5) self-efficacy, and (6) cues to action [23]. According to the HBM, people’s beliefs about their risk and their perception of the benefits of taking action to avoid it, influence their readiness to take action [15, 21–22, 24]. Using the HBM, health behavior can be modified positively if the 6 posits are perceived by the person [23]. According to the results of our study, taxi drivers that participated in our study, do not perceive the severity of their risk. Participants admitted that they go to the doctor and start paying attention to their health condition only when they get seriously sick. Another posit of the HBM, understanding benefit of actions, is also not perceived by taxi drivers. Participants understand that they should be involved in physical activity, but do not pursue physical activity. They stated that they are too busy and tired to exercise daily without realizing the benefits of having a healthy life style. Findings from the focus groups also demonstrate that taxi drivers do not possess self-efficacy, as they are not confident that they are able to change their own health behavior. They openly admitted to having poor discipline, lack of organizational skills, and lack of time management skills. But, they expressed their wish to get information about time management, healthy snacks, places where they can get affordable and healthy food, learn more about different physical activities, and places where they can exercise. The sixth posit of the HBM model is the cues for action which should trigger the action to change behavior. Cues such as physical pain or illness in them or family members of cab drivers, trigger a visit to the physician’s office. Cab drivers were open to receiving educational material provided by physicians or health information provided on TV/cellphone about disease prevention. DISCUSSION/SIGNIFICANCE OF IMPACT: Obesity is steadily on the increase in the US population and has become a major public health concern [1–3]. Latinos are at the higher risk of heart diseases such as obesity, hypertension compared to other ethnical groups [3, 13]. There is a higher prevalence of obesity among particular occupational groups with cab drivers having one of the highest obesity prevalence among all professions [5, 7–9, 13]. Obesity risks therefore seem to affect NYC cab drivers who are of Latino background more than others. Surveys conducted in different countries in Asia, Europe, and Africa reported that taxi, truck, and bus show that drivers are at a higher risk of developing obesity, diabetes, and hypertension [5, 8–11]. This study is the first to evaluate the knowledge, perception, and behaviors of NYC Latino taxi cab drivers with respect to obesity. The study uncovers factors and barriers that contribute to their behavior, and identify possible ways that can modify their behavior and decrease their chances of developing obesity. The study results demonstrated that Latino immigrant taxi drivers perceive themselves at a high risk for obesity development. As the result of discussions with focus groups, the eight dominant themes were identified. Participants perceive their risk susceptibility and understand that working as a driver is a sedentary occupation with lack of physical activity significantly contributing to obesity development. Additionally, taxi drivers report that their unhealthy diet is a major factor that contributes to their weight gain. Taxi drivers perceive their poor diet as the result of the food they consume being high in fat content. Due to financial constraints and their cultural diet requirements, they feel limited to unhealthy food options. They acknowledge the risk that poor diet contributes to obesity, high cholesterol, obesity development. Participants also expressed that work stress is another important factor. Busy traffic, lack of organization, financial stress to support their families-push them to work prolonged hours. Participants also admitted that in their leisure time, they use alcohol, smoke cigarettes, and watch TV, instead of going to the gym, because they feel too tired to exercise. Taxi drivers perceive their barriers as a lack of education and knowledge about healthy food choices, places where they can buy healthy affordable snacks, information about physical activities, stress management skills, and organizational skills. Other perceived barriers that prevent them from leading healthy lifestyle include lack of discipline, lack of time for physical activity, economic uncertainty, financial responsibility and the perception that the wellbeing of their families is more important than themselves and their health. HBM is a widely used model that helps to identify perception of risks of unhealthy behavior, barriers to healthy behavior, actions taken by patients to stay healthy, self-efficacy, and commitment to goals. Based on the Glasgow theory, the core of health behavior models is the identification of the barriers and self-efficacy [25]. Our study is unique as it involves using the HBM to explain the basis of taxi cab drivers’ behavior. Results of our research study showed that our participants perceived barriers very well. However, lack of self-efficacy, lack of perceiving benefits of action, lack of cues to action, and lack of understanding the risk of disease severity explain why taxi drivers have greater risk for obesity among occupations, and are not ready to embrace health behavior modification. This qualitative study shows us where the window of opportunity for intervention lies, how we can intervene and modify the health behavior of the at-risk NYC Latino cab driver population. By Glasgow theory, self-efficacy is an important factor in behavior modification models [25]. If the barriers that are perceived by participants as too high, and self-efficacy is low, one can intervene by improving self-efficacy. Bandura has offered ways to increase patients’ self-efficacy by using three strategies: (a) setting small, incremental, and achievable goals; (b) using formalized behavioral contracting to establish goals and specify rewards; and (c) monitoring and reinforcement, including patient self-monitoring by keeping records [20]. We can also improve perception of the benefits of action by providing cues to action namely education during the office visits, by providing reading materials, and the use of modern technology (emails, interactive Web sites, apps, etc.). A study was conducted in South Asia, encouraging taxi drivers to exercise through the use of pedometers [7]. This study provides an example of ways to motivate taxi drivers, improve their self-efficacy, overcome barriers, and provide cues to action. As one of the theories that can explain and help in behavioral modification, the Health Belief model includes the impact of the environment and elements of social learning. Using this model, we were able to differentiate and identify the factors that influence their behavior that need to be addressed by health care workers and public health representatives to improve obesity related risks among inner city taxi cab drivers in NYC.

